# Taxonomic revision of the genus *Aberdareleria* (Diptera, Heleomyzidae, Oecotheini) with description of a new species from China

**DOI:** 10.3897/zookeys.1278.167489

**Published:** 2026-04-30

**Authors:** Wei Zeng, Dawei Hong, Ding Yang

**Affiliations:** 1 Department of Entomology, College of Plant Protection, China Agricultural University, Beijing 100193, China China Agricultural University Beijing China; 2 College of Plant Science, Xizang Agricultural and Animal Husbandry University, Nyingchi, Xizang 860000, China Xizang Agricultural and Animal Husbandry University Nyingchi China

**Keywords:** *
Aberdareleria
*, disjunct distribution, generic key, Heleomyzidae, Heleomyzinae, Oecotheini, species key, taxonomy, vicariance

## Abstract

The genus *Aberdareleria* Woźnica, 1993 (Diptera: Heleomyzidae) was previously considered monotypic and endemic to the Aberdare Mountains, Kenya. Here, we describe *A. qinghaiensis***sp. nov**. from Qinghai Province, China, marking the first record of the genus outside the Aberdare Mountains in Kenya and revealing a remarkable Afrotropical-Palearctic disjunction (>6000 km). The new species differs significantly from *A. freidbergi*, in traits such as having reduced setae on the mid tibia (only anterodorsal and posterodorsal setae) and only spine-like setae on the female cercus (absence of hair-like setae). Through a taxonomic revision of the genus, we provide a diagnostic key, detailed morphological comparisons, and discuss the biogeographic implications of this transcontinental distribution, suggesting either ancient vicariance or avian-mediated dispersal. This discovery highlights the underestimated diversity of high-altitude Diptera in the Qinghai-Tibet Plateau.

## Introduction

The family Heleomyzidae comprises over 700 described species worldwide, many of which are associated with decaying organic matter or small mammal nests (Scudder and Cannings 2006; Woźnica and Kirk-Spriggs 2021; Weele et al. 2023). Many species exhibit diverse adaptations to extreme environments, including high-altitude ecosystems and subterranean habitats (Block 2002; Hågvar and Greve 2003; Soszyńska-Maj and Woźnica 2016). The tribe Oecotheini Gorodkov, 1972 within subfamily Heleomyzinae is particularly notable for its specialized ecological adaptations, often inhabiting burrows or artificial underground environments (Papp 1981).

Within the tribe Oecotheini, the genus *Aberdareleria* Woźnica, 1993 has been considered an endemic monotypic genus, known only from the type species, *A. freidbergi*, in the Aberdare Mountains (3000–4000 m), Kenya (Woźnica and Kirk-Spriggs 2021). Its specialized morphology (e.g., sclerotized female cercus) suggests a long history of isolation, a hypothesis that is strongly supported by the highly apomorphic nature of these traits, particularly a female cercus that is so distinctively and strongly sclerotized that it constitutes an autapomorphy within the tribe. Such profound structural specialization, combined with a distribution restricted to a single Afrotropical montane system, most likely reflects an ancient vicariance event followed by prolonged evolution in allopatry. Nevertheless, the absence of congeneric species in other continents has left its phylogenetic position uncertain (Gorodkov 1972; Woźnica 1993). Previously, this tribe included three other genera: *Oecothea*, *Eccoptomera*, and *Pseudoleria* (Woźnica 1993). The classification of *Aberdareleria* within Oecotheini is firmly supported by its possession of key synapomorphies that define the tribe, such as specific antennal structures. This confirms the tribe’s morphological cohesion as outlined by Gorodkov (1972). Moreover, the extreme autapomorphies present in *Aberdareleria* do not conflict with the tribal diagnosis. Instead, they demonstrate the considerable morphological disparity and evolutionary potential that can develop within a monophyletic lineage over an extended period. The unique structure of the female cercus and spermatheca in *Aberdareleria* provides crucial evidence for morphological diversity within the tribe and supports the tribal classification system of Gorodkov (1972).

Species in the tribe Oecotheini are generally closely associated with the nests or burrows of small mammals (Papp 1981), and some species are adapted to artificial underground spaces (such as mines and quarries), reflecting specialized ecological adaptations. Currently, *Aberdareleria* is recorded only in the high-altitude areas (3000–4000 m) of the Aberdare Mountains in Kenya, and its ecological habits are not yet clearly understood (Woźnica 1993). Morphological characters of *A. freidbergi*, such as its sclerotized female terminalia, suggest that it likely inhabits small mammal (rodent) burrows. In these microhabitats, its saprophagous larvae presumably develop, and the species has been reported as a cryptic resident of moorlands in montane areas (Woźnica and Kirk-Spriggs 2021).

Disjunct distributions between African and Asian high-altitude Diptera (e.g., *Suillia*, *Trixoscelis*) are often attributed to either Gondwanan vicariance or recent dispersal (McAlpine 1984; Papp 1999). However, such patterns remain poorly documented in Heleomyzidae, particularly for taxa with limited dispersal ability. The discovery of *Aberdareleria* in the Qinghai-Tibet Plateau (3491 m) challenges the assumption of its African endemism and provides a unique opportunity to test biogeographic hypotheses through comparative morphology.

This study aims to describe *A. qinghaiensis* sp. nov. as the second species of the genus, revise the generic diagnosis of *Aberdareleria*, and evaluate potential mechanisms underlying its transcontinental distribution.

## Material and methods

Specimens were examined and illustrated using a ZEISS Stemi 2000-C stereomicroscope. For genitalic examination, the apical segments of the abdomen were macerated in a heated 85% lactic acid solution for approximately 50 mins. Upon completion of the dissection, the specimens were transferred to fresh glycerol and stored in microvials, which were then pinned alongside their corresponding specimens. In this study, a female specimen collected from Xianmi, Menyuan County, Qinghai Province, on 30 July 2020 was examined. The specimens examined during this study have been deposited in the State Key Laboratory of Agricultural and Forestry Biosecurity, MARA Key Laboratory of Surveillance and Management for Plant Quarantine Pests, College of Plant Protection, China Agricultural University (**CAU**), Beijing 100193, China. Morphological terminology for adult structures primarily follows the framework established by Cumming and Wood (2017). The distribution map was generated by https://www.bioinformatics.com.cn (last accessed on 6 Aug 2025), an online platform for data analysis and visualization.

## Taxonomy

### Genus Aberdareleria

Taxon classificationAnimaliaDipteraHeleomyzidae

Woźnica, 1993

897BB819-7534-5F9B-9D6F-C85FD850009A

Aberdareleria Woźnica, 1993: 59.

#### Type species.

*Aberdareleria freidbergi* Woźnica, 1993 (monotypic).

#### Revised diagnosis.

One pair of fronto-orbital setae. Arista long, with short-pubescent. Thorax with four pairs of dorsocentral setae (one presutural and three postsutural), two notopleural setae, and one well-developed katepisternal seta. Scutellum covered with numerous small setulae in addition to its major scutellar setae; thorax with bare pleura except katepisternal seta. Mid tibia with two or multiple rows of setae (anterodorsal, posterodorsal, anteroventral, and posteroventral in *A. freidbergi*, but reduced to only anterodorsal and posterodorsal rows in *A. qinghaiensis*). Wing membrane lightly tinged, and costa with well-developed spines; vein A_1_+CuA_2_ weakly extended to margin; female cercus highly sclerotized with spiny setae, spermathecae spherical with sclerotized base (Woźnica 1993), and with a small apical appendix or tubercle.

#### Differentiation from *Oecothea*.

The genus *Aberdareleria* can be distinguished from *Oecothea* by the following unique combination of characters: the 9^th^ sternite well developed, strongly sclerotized (9^th^ sternite small, simple, membranous or weakly sclerotized in *Oecothea*), the strongly sclerotized female cercus bearing spiny setae (hairy setae only in *Oecothea*), the spermathecae with a sclerotized base and a small apical appendix/tubercle (entirely membranous or uniformly sclerotized without appendix in *Oecothea*).

##### Key to species of the genera of Oecotheini

Modified Woźnica 1993.

**Table:** 

1	Mid tibia with several setae in the middle part	**2**
–	Mid tibia without seta in the middle part	**3**
2	Female cercus with spiny setae; 9^th^ sternite well developed, strongly sclerotized	***Aberdareleria* Woźnica, 1993**
–	Female cercus with hairy setae; 9^th^ sternite small, simple, membranous or weakly sclerotized	***Oecothea* Haliday, 18373**
3	Mid femur with rows of bristles anteriorly, anepimeron with setae	***Pseudoleria* Garrett, 1921**
–	Mid femur, rows of bristles anteriorly, anepimeron without setae	***Eccoptomera* Loew, 1862**

##### Key to species of the genus *Aberdareleria* Woźnica, 1993

Only female.

**Table:** 

1	Mid tibia with anterior and posterior dorsal, anterior ventral and ventral setae; 9^th^ tergite very narrow and ring-shaped; 9^th^ sternite broad; cercus setae spiny and hair-like setae	***A. freidbergi* Woźnica, 1993**
–	Mid tibia only with anterior and posterior dorsal setae; 9^th^ tergite rectangular, approximately 0.5 times as long as wide; 9^th^ sternite slightly constricted medially, posterior half densely covered with small setulae; cercus with only spiny setae, but without hair-like setae	***A. qinghaiensis* sp. nov**.

### Aberdareleria qinghaiensis sp. nov.

Taxon classificationAnimaliaDipteraHeleomyzidae

AD6782CA-AFEB-5BF8-83D6-2C3C1CE5FCC5

https://zoobank.org/DDEBD5A1-B7F0-4DFD-B33B-1D77B753E21F

#### Diagnosis.

Eye round, cheek-eye ratio about 1.23. Mesonotum concolorous with postpronotum. Mesonotum pale yellow, with a very fine, indistinct light brown stripe medially; incomplete light brown stripe between first and last pairs of dorsocentral setae, gradually converged medially near last pair of dorsocentral setae; a pair of faint light yellowish-brown stripes laterally postsuturally outside dorsocentral rows. Scutellum covered with small black setulae. Pleura concolorous with dorsum, except anepisternum almost entirely yellowish brown. Wing veins yellow on upper surface, slightly darker below, without distinct clouding around veins. Mid tibia only with anterior and posterior dorsal setae. 9^th^ tergite rectangular, approximately 0.5 times as long as wide. Cercus with only spiny setae.

#### Description.

**Female**. Body length 6.2 mm, wing length 6.8 mm.

**Figures 1, 2.**
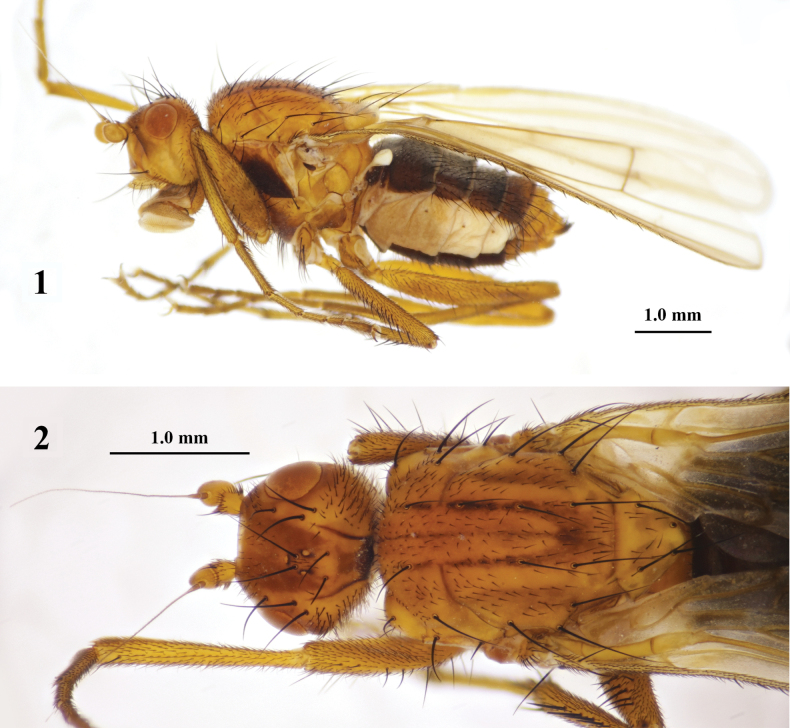
*Aberdareleria qinghaiensis* sp. nov., female holotype. Habitus, lateral view (**1**). Head and mesonotum, dorsal view (**2**).

***Head*** (Figs 1, 2) covered with pale yellowish white pruinescence; frons dull pale yellowish, with symmetrical light orange-brown patches surrounding ocellar triangle posteriorly; occiput light orange-brown; parafacial angle light yellowish brown; gena not distinctly paler, postgenal area dull pale yellow; eyes light red. Head in lateral view approximately 1.28 times as high as wide; eyes rounded; genal height to eye height ratio 1.23. Frontal orbits nearly parallel to eye margin; one fronto-orbital seta present. Small setulae scattered from anterior frons to mid-posterior frons. Antennal scape and pedicel dull pale yellow; both scape and pedicel with marginal apical setae; pedicel with one longer dorsal seta slightly inclined inward and long ventral marginal setae; first flagellomere dull yellow, covered with light yellowish-brown pubescence from apex to middle; arista light yellowish brown, basally swollen, with short pubescence, 7.45 times as long as first flagellomere. One well-developed vibrissa present; peristomal setae slightly longer than first row of postgenal setae, arranged in one relatively regular single row; area along peristomal setae and lower margin of peristome light yellowish brown. Palpus pale yellow, apically enlarged, with longest dorsal setae near apex medially; proximal dorsal area with slightly dense small setulae, remaining areas with small sparse setulae. Proboscis pale grayish yellow, covered with light brown setulae.

***Thorax*** (Figs 1, 2) with pale yellow to light orange-yellow, covered with pale yellowish white pruinescence. Mesonotum pale yellow with one very narrow, indistinct light brown stripe medially; one incomplete light brown stripe between rows of dorsocentral setae from first to last pair, gradually converging medially near last pair of dorsocentral setae; one pair of faint light yellowish-brown stripes laterally postsuturally outside dorsocentral rows. Postpronotum concolorous with mesonotum. Postpronotum with one well-developed postpronotal seta and ten small setulae. Four dorsocentral setae present (one presutural, three postsutural); five to six irregular rows of acrostichal setulae between dorsocentral rows. Two notopleural setae present, anterior one longer than posterior. Scutum without distinct prescutellar setae. Scutellum slightly paler than mesonotum, with light gray spots near mid-lateral areas; apex slightly blunt; dorsal surface covered with small black setulae. Pleura concolorous with dorsum, except anepisternum almost entirely yellowish brown. Proepisternum with one well-developed seta and five tiny setulae; proepimeron with five tiny setulae. One well-developed katepisternal seta present; right katepisternum with one additional weaker anterior seta (1/5 length of posterior seta; absent on left side), surrounded by several tiny setulae. Remaining pleural areas without setulae.

***Wing*** (Fig. 4) slightly tinged light yellowish brown; veins yellow on upper surface, slightly darker below; veins light yellowish brown. Crossveins not distinctly darkened, without clouding around them. Veins A_1_+CuA_2_ weakly extended to margin. Costal spines arranged in alternating long and short pattern. Halter pale, whitish-brown, knob paler.

**Figures 3–5.**
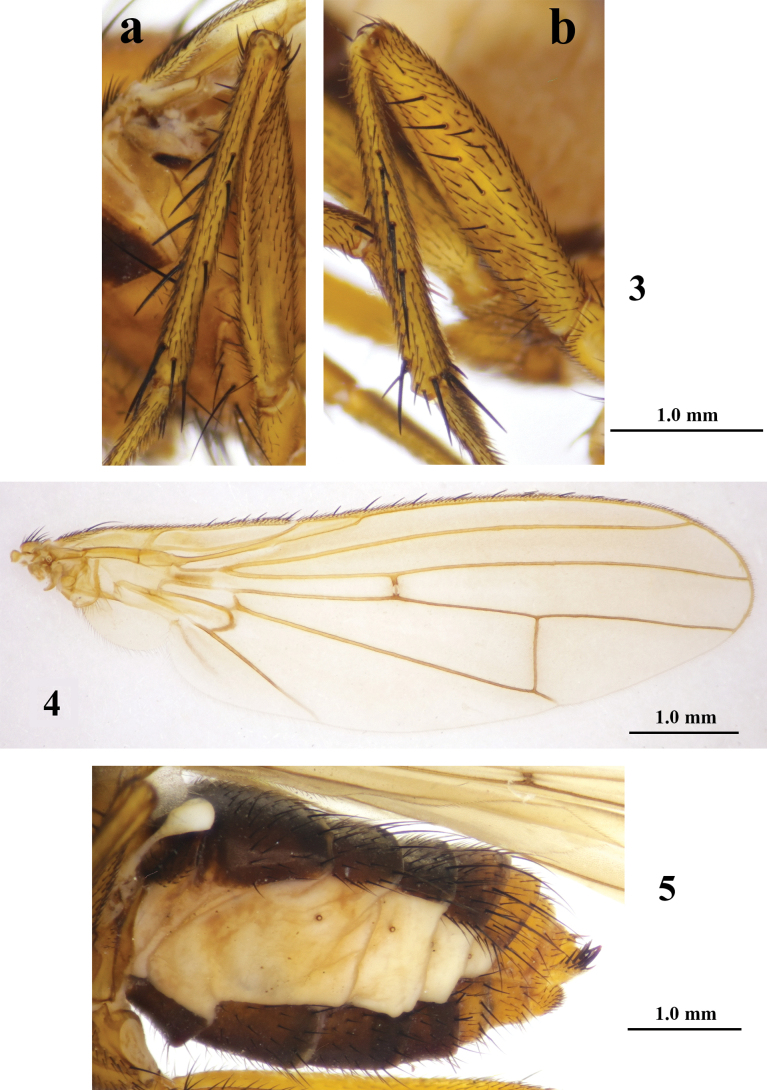
*Aberdareleria qinghaiensis* sp. nov. Mid femur and mid tibia (**3**), mid tibia in dorsal view (**a**), mid femur and mid tibia in frontal view (**b**). Wing (**4**). Abdomen, lateral view (**5**).

***Legs*** (Figs 1, 2, 3) dull yellow, all tarsi unmarked. Fore femur with dorsal setae and ventral setae from base to apex. Mid femur with anterodorsal setae from base, near apex, becoming more developed near apex; anteroventral setae from base to middle; anterior spinose setae from middle to apex; one posterior apical seta. Hind femur with dorsal setae near apex directed backwards and anteroventral setae. Fore and hind tibiae with dorsal preapical seta; mid tibia with setae at near apex and apex. Fore tibia apically with cluster of yellowish-brown ventral setae, brush-like. Mid tibia with setae medially (left: 4 anterodorsal and 3 posterodorsal; right: 5 anterodorsal and 2 posterodorsal). All legs with fourth tarsomere slightly shorter than fifth; fore and hind tarsomere 1 with light yellowish-brown brush-like ventral setae longer near apex.

**Figures 6–8.**
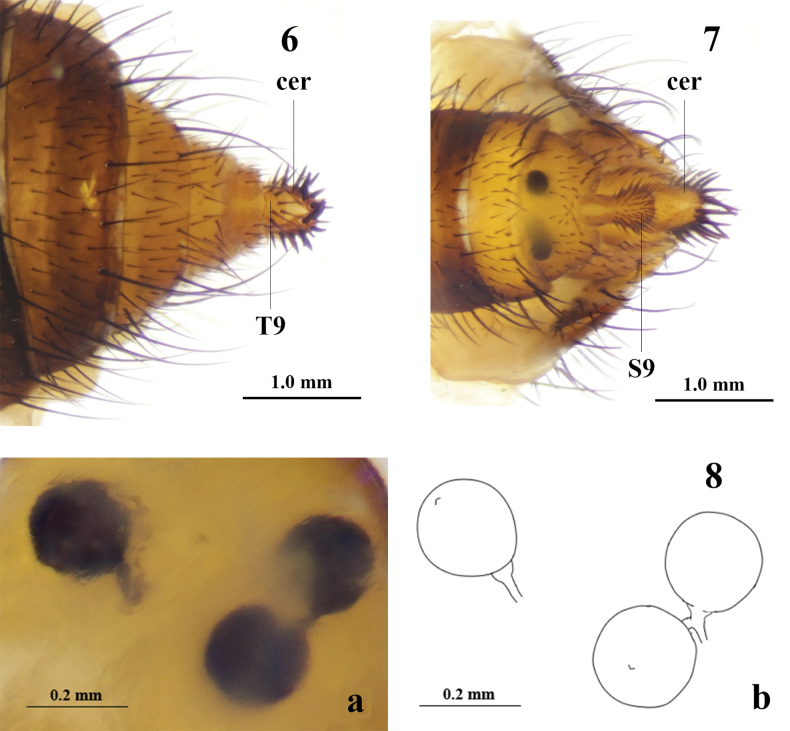
*Aberdareleria qinghaiensis* sp. nov., postabdomen. Female terminalia in dorsal (**6**), ventral (**7**). Spermatheca (**8**), (**a**: photograph), (**b**: drawing). Abbreviations: cer = cercus (cerci), T9 = tergite 9, S9 = sternite 9.

***Abdomen*** (Figs 5, 6, 7): Tergites 1–5 and sternites 1–4 light yellowish brown, remaining segments yellow. Tergite 2 1.5 times as long as tergite 3. Tergites 2–6 with apical marginal setae.

***Female terminalia*** (Figs 6–8): Yellow to light yellowish brown. Tergite 9 rectangular, 0.5 times as long as wide, with spine-like setae. Sternite 9 well developed, slightly constricted medially, posterior half densely covered with small setulae. Cercus extremely short and robust, fused medially, with developed spine-like setae densely covering dorsal, apical and lateral surfaces, without hair-like setae. Spermathecae dark brown, well sclerotized: one single, other two grouped together; nearly spherical, slightly constricted near base, with small apical tubercles.

**Male**. Unknown.

#### Type material.

***Holotype*** • ♀, China, Qinghai, Menyuan, Xianmi (37°31.8'N, 101°34.8'E), 3491 m, 2020.VII.30, Zhenning Chen (CAU).

#### Other material examined.

None.

#### Distribution.

China (Qinghai).

#### Etymology.

The specific name refers to the type locality, Qinghai.

##### Comparative morphology based on Woźnica (1993)

The newly described *A. qinghaiensis* can be clearly distinguished from the type species of the genus, *A. freidbergi*, by the following characters (based on the original description by Woźnica (1993)):

##### Diagnosis of *Aberdareleria freidbergi* Woźnica, 1993

Eye elliptical, cheek-eye ratio about 0.75. Mid tibia with 10–12 (anterodorsal + posterodorsal + anteroventral + ventral) setae. 9^th^ tergite narrow and ring-shaped. Cercus with spiny and hair-like setae.

**Comparative notes**.

**Female. *Colouration***: General body colouration also differs; *A. qinghaiensis* has a pale yellow mesonotum with faint stripes, while the mesonotum of *A. freidbergi* is described as slightly darkened.

**Figures 9, 10.**
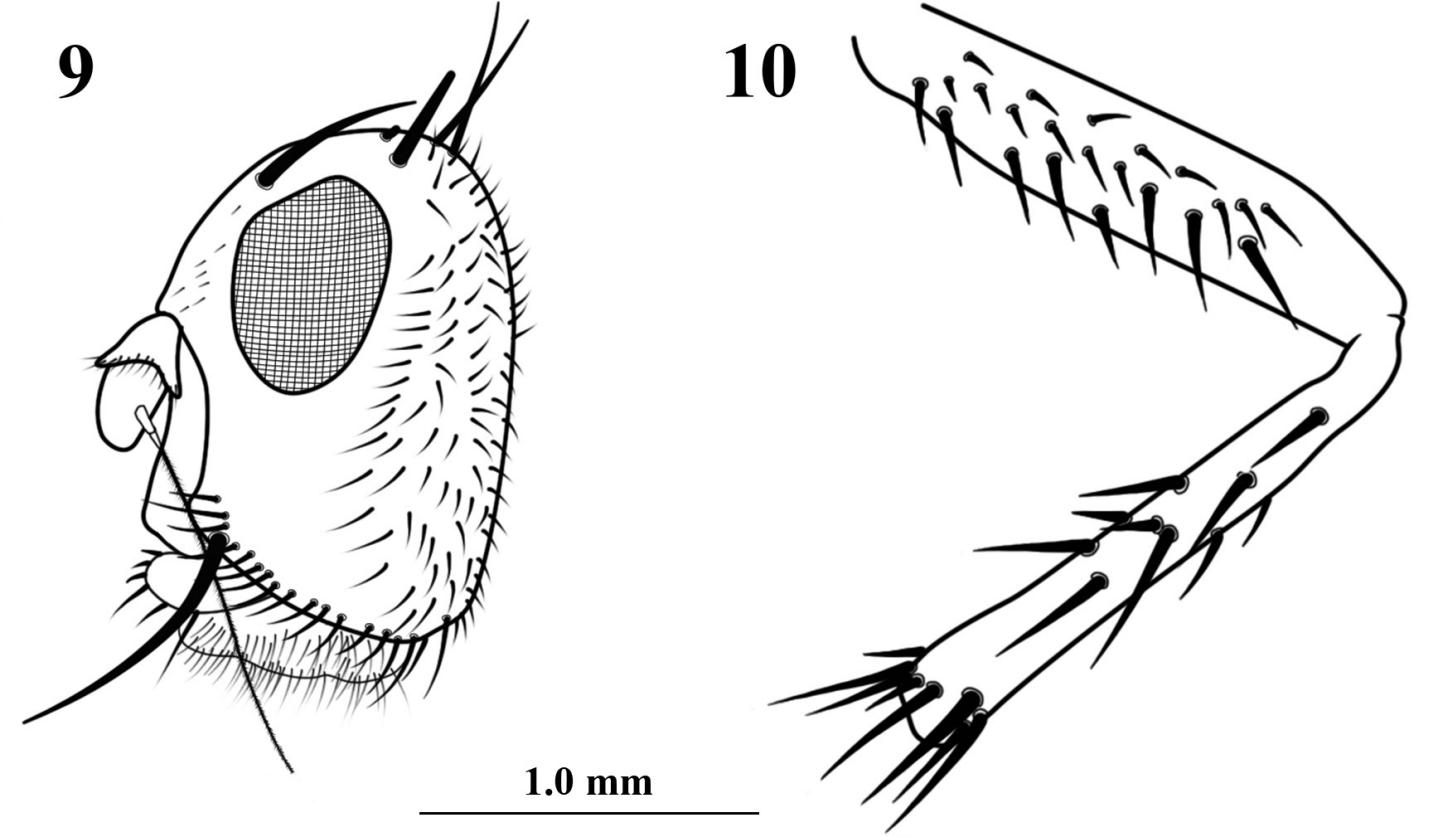
*Aberdareleria freidbergi*, female holotype. Head, lateral view (**9**). Mid femur and tibia, lateral view (**10**) (line drawing modified from Woźnica 1993: figs 1, 2).

***Head*** (Figs 1, 2, 9): The most conspicuous difference lies in the eye shape. In *A. qinghaiensis*, the eye is rounded, whereas it is described as elliptical in *A. freidbergi*. This results in a different genal height to eye height ratio: approximately 1.23 in *A. qinghaiensis*, compared to a ratio in which the genal height and eye height are nearly equal in *A. freidbergi*. Furthermore, the arista of *A. qinghaiensis* is 7.45 times as long as the first flagellomere, which is proportionally longer than that of *A. freidbergi* (5.30 times as long).

***Thorax***: The pleura concolorous with the mesonotum, excluding the anepisternum in *A. qinghaiensis*, whereas the original description of *A. freidbergi* does not mention this pattern, suggesting a potential difference.

***Wings***: The wing crossveins in *A. qinghaiensis* are slightly clouded. The original description of *A. freidbergi* notes the same presence of the crossveins.

***Legs*** (Figs 1, 2, 3, 10): The chaetotaxy of the mid tibia provides a key diagnostic character. In *A. qinghaiensis*, setae are present only on the anterodorsal and posterodorsal surfaces (with 4–5 and 2–3 setae, respectively). In contrast, the mid tibia of *A. freidbergi* bears a more extensive complement of 10–12 setae, including rows on the anteroventral and ventral surfaces in addition to the dorsal rows.

**Figures 11, 12.**
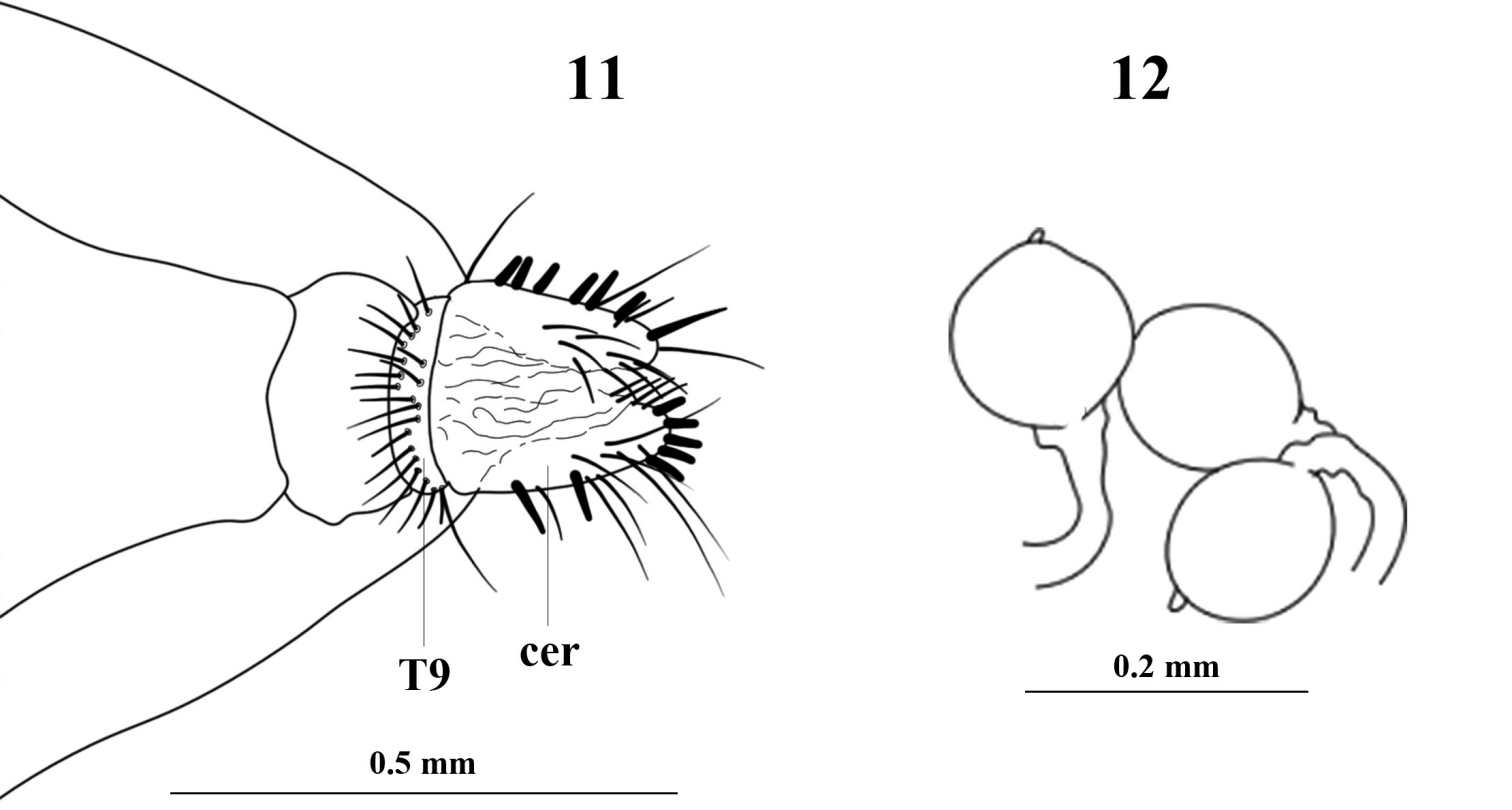
*Aberdareleria freidbergi*, female holotype. Postabdomen, dorsal view (**11**). Spermatheca (**12**) (line drawing modified from Woźnica 1993: figs 5, 6). Abbreviations: cer = cercus (cerci), T9 = tergite 9.

***Female terminalia*** (Figs 6–8, 11, 12): Significant differences are observed in the female terminalia. The 9^th^ tergite is rectangular and approximately 0.5 times as long as wide in *A. qinghaiensis*, but is described as narrow and ring-shaped in *A. freidbergi* with broad 9^th^ sternite and *A. qinghaiensis* with 9^th^ sternite slightly constricted medially. The setation of the cercus also differs: it bears only spiny setae in the new species, while in *A. freidbergi* the cercus possesses both spiny and hair-like setae.

***Shared generic synapomorphies***: Despite these specific differences, both species unequivocally share the critical synapomorphic characters that define the genus *Aberdareleria*. These include the highly sclerotized female cercus and the spermathecae, which are dark brown, well sclerotized, nearly spherical, and possess both a sclerotized base and a small apical tubercle (Woźnica 1993; observed in *A. qinghaiensis*).

## Discussion

### Generic status of *Aberdareleria* and systematic placement of *A. qinghaiensis* sp. nov.

The discovery of *A. qinghaiensis* presents a taxonomic challenge, as its morphology, particularly the reduced mid-tibial chaetotaxy and cercus with only spiny setae deviates from the diagnosis of *Aberdareleria* established solely on the single female holotype of *A. freidbergi* (Woźnica 1993). This raises the critical question of its generic assignment: does it belong to *Aberdareleria*, or does it indicate a closer relationship to *Oecothea*?

We argue for its inclusion in *Aberdareleria* based on an hierarchical assessment of taxonomic characters. The highly specialized and sclerotized structures of the female terminalia are widely regarded as phylogenetically conservative and reliable for generic delineation. In this light, *A. qinghaiensis* shares two decisive, likely synapomorphic conditions with *A. freidbergi* that are absent in *Oecothea*: a well-developed, strongly sclerotized 9^th^ sternite and sternite and a cercus armed with robust spine-like setae. This stands in clear contrast to the female terminalia of *Oecothea* species, which are characterized by weakly sclerotized sternites VIII–IX and a cercus bearing only hair-like setulae (Gorodkov 1959).

The divergent mid-tibial chaetotaxy, while a significant specific difference, may be more labile. This character could be susceptible to ecological adaptation for locomotion in specific substrates and thus variable at the species level. The original generic diagnosis by Woźnica (1993), based on a single specimen, might have overemphasized this feature. The condition in *A. qinghaiensis* likely represents intrageneric variation rather than grounds for exclusion.

It is noteworthy that the presence of spine-like setae on the cercus may not be a definitive generic character in all heleomyzid taxa. Our reasoning is that cercal setation type can vary within a genus. For instance, in the genus *Anorostoma*, the chaetotaxy of the female cercus varies between species: some species have hairs while others have spines. (However, this genus itself is considered in need of revision, with some species thought to be closer to *Neoleria* (Papp, 1998). Thus, the current taxonomic uncertainty surrounding *Anorostoma* may limit its strength as a model for character evolution in other genera.) Therefore, cercal setation should not be used as the sole criterion for generic delineation. However, it retains practical diagnostic value when combined with other characters, as seen in keys distinguishing *Acantholeria* from *Schroederella* (Papp, 1998). Most crucially, our argument for the validity of *Aberdareleria* does not rest on a single character but on a consistent suite of derived traits: the unique and strongly sclerotized structural complex of the entire female terminalia (cercus, sternite 9, and spermathecae), which is shared by both known species and is absent in *Oecothea*.

**Figure 13.**
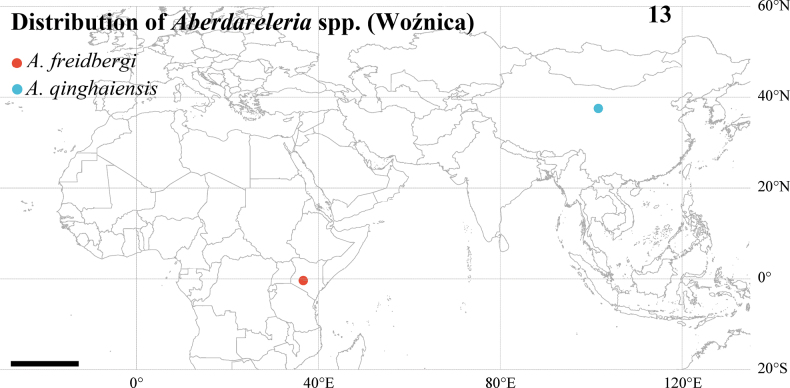
*Aberdareleria* spp., distribution map (scale: 1000 km). Red circle: *A. freidbergi* (Aberdare Mountains, Kenya); blue triangle: *A. qinghaiensis* sp. nov. (Qinghai, China). Base map from Natural Earth.

The genus *Aberdareleria* thus exhibits a remarkably disjunct distribution between East Africa and the Qinghai-Tibet Plateau (Fig. 13). *Aberdareleria freidbergi* is endemic to the Aberdare Mountains, Kenya, while *A. qinghaiensis* is found exclusively in the high-altitude grasslands of Qinghai, China. As detailed in the section Comparative morphology based on Woźnica (1993), the key morphological differences between the two species lie in the chaetotaxy of the mid tibia and the setation of the female cercus, which serve as crucial characters for species identification.

### Morphological distinctions and biogeographic implications

The Afrotropical-Palearctic distribution of *Aberdareleria* shows a biogeographic pattern similar to that seen in other dipteran groups. Two hypotheses are worth considering: Firstly, ancient distribution across warmer Paleogene/Pleistocene habitats, with subsequent extinction in intermediate areas due to aridification. Secondly, recent dispersal via migratory birds or other zoochorous mechanisms.

It is crucial to distinguish between the morphological basis for our current taxonomic decision and the evolutionary hypotheses that remain to be tested. Our placement of *A. qinghaiensis* within *Aberdareleria* is firmly grounded in the shared, derived complex of female terminalia characters, a suite of traits generally considered reliable for generic delineation in Diptera. This decision is independent of and antecedent to determining the evolutionary mode (homology vs. parallel evolution) underlying the remarkable ecology of the species and the morphological similarities across continents. The resolution of this evolutionary question awaits molecular phylogenetic testing.

Both known species of *Aberdareleria* are specialized for high altitude environments (3000–4000 m in Kenya, 3491 m in Qinghai). The remarkable convergence of the two *Aberdareleria* species in high-altitude niches, despite their vast geographic separation, invites deeper evolutionary inquiry. Future molecular phylogenies will be crucial for interpreting this pattern. If they confirm the genus’s monophyly, these shared alpine adaptations would most parsimoniously be interpreted as homologous traits, indicating a common ancestor already adapted to montane conditions. Conversely, if the two species are found to belong to separate lineages within *Oecothea*-like ancestors, their morphological similarities would instead constitute a striking case of parallel evolution. Such a finding would directly challenge the current generic delineation, prompting a necessary taxonomic revision. Our taxonomic decision to include *A. qinghaiensis* within *Aberdareleria* is based on the most conservative morphological evidence currently available: the shared, derived, and highly specialized complex of female terminalia characters (strongly sclerotized ninth sternite and cercus with spine-like setae). In this context, it is worth considering that if *A. qinghaiensis* had been discovered first, its possession of this highly specialized terminalia complex would likely have warranted establishing a new genus distinct from *Oecothea*. The subsequent discovery of *A. freidbergi* would then be viewed as a congeneric species exhibiting more developed mid-tibial chaetotaxy. This perspective reinforces our interpretation that the difference in tibial chaetotaxy represents specific-level variation, rather than a challenge to the generic unity defined by the conserved terminalia. This does not preclude alternative evolutionary histories for these traits. The remarkable convergence of the two *Aberdareleria* species in high-altitude niches, despite their vast geographic separation, underscores the critical need for molecular phylogenetic studies. Such data are essential to definitively determine whether their similar phenotypes related to high-altitude adaptation stem from a single adaptation in a common ancestor (homology) or from parallel evolution under similar selective pressures after isolation. Thus, the disjunct distribution of *Aberdareleria* offers a compelling framework for testing these contrasting evolutionary scenarios.

The disjunct distribution of *Aberdareleria* (Africa or Asia) parallels patterns observed in other heleomyzids groups, suggesting possible ancient dispersal via warmer Paleogene corridors. Alternatively, the recent long-distance dispersal by migratory birds could explain this pattern, as avian-mediated dispersal is strongly suggested by the biology of heleomyzid flies in the genus *Neossos*, whose life cycle is obligately associated with bird nests, indicating a clear dispersal pathway via avian hosts (Gilbert and Wheeler 2007). Human activities can also drive similar disjunctions, as documented by the spread of the genus *Pseudoleria* from the Nearctic to Africa and Australia (McAlpine 1984), a typical example of human-mediated isolated distribution. Additionally, the *Trixoscelis* is widely distributed across arid regions worldwide (Cogan 1977), *Prosopantrum* exhibits a disjunct distribution between Africa and South America (Papp 1999), and *Suillia* is widely distributed in the Northern Hemisphere, with a few species invading high mountains in Africa (Woźnica 2012). These distribution patterns reflect the influence of ecological adaptation, the breakup of the Gondwanan supercontinent, and glacial-period relicts. These cases demonstrate that the isolated distributions in Heleomyzidae result from multiple overlapping factors, providing important material for the study of insect biogeography.

### Conclusions and future prospects

The discovery of *A. qinghaiensis* in China challenges the previous assumptions about the restricted distribution of this genus and provides new insights into the historical biogeography of high-altitude Diptera. The Qinghai-Tibet Plateau is a biodiversity hotspot, and this discovery underscores the need for further surveys in understudied alpine ecosystems.

This study is based solely on female specimens. Future collections of males and molecular data (e.g., COI barcoding) will be important for confirming species boundaries and estimating divergence times.

## Supplementary Material

XML Treatment for Aberdareleria

XML Treatment for Aberdareleria qinghaiensis

## Additional information

### Use of AI

No use of AI was reported.

### Author ORCIDs

Wei Zeng https://orcid.org/0000-0001-8749-053X

Dawei Hong https://orcid.org/0000-0003-4645-3809

Ding Yang https://orcid.org/0000-0002-7685-3478

### Data availability

The data underpinning the analysis reported in this paper are deposited at GBIF, the Global Biodiversity Information Facility, and are available at: https://www.gbif.org/dataset/d1cae9ed-8d4f-44d0-add0-b5468779de0f.
